# Impact of CDT Toxin on Human Diseases

**DOI:** 10.3390/toxins8070220

**Published:** 2016-07-15

**Authors:** Tiphanie Faïs, Julien Delmas, Arnaud Serres, Richard Bonnet, Guillaume Dalmasso

**Affiliations:** 1 Microbes, Inflammation, Intestin et Susceptibilité de l’Hôte (M2iSH), Clermont Université, Université d’Auvergne; INSERM U1071; INRA USC2018, Clermont-Ferrand 63000, France; jdelmas@chu-clermontferrand.fr (J.D.); rbonnet@chu-clermontferrand.fr (R.B.); 2 Laboratoire de Bactériologie, Centre Hospitalier Universitaire, Clermont-Ferrand 63000, France; aserres@chu-clermontferrand.fr; 3 Institut Universitaire de Technologie, Université d’Auvergne, Aubière 63170, France

**Keywords:** CDT toxin, CDT-producing bacteria, DNA damage

## Abstract

Cytolethal distending toxin (CDT) is found in Gram-negative bacteria, especially in certain *Proteobacteria* such as the *Pasteurellaceae* family, including *Haemophilus ducreyi* and *Aggregatibacter (Actinobacillus) actinomycetemcomitans*, in the *Enterobacteriaceae* family and the *Campylobacterales* order, including the *Campylobacter* and *Helicobacter* species. In vitro and in vivo studies have clearly shown that this toxin has a strong effect on cellular physiology (inflammation, immune response modulation, tissue damage). Some works even suggest a potential involvement of CDT in cancers. In this review, we will discuss these different aspects.

## 1. Introduction

The cytolethal distending toxin (CDT), produced by several Gram-negative pathogenic bacteria, belongs to the AB toxin family. The AB_2_ trimer is thus composed of an active subunit (CdtB) and two binding subunits (CdtA and CdtC). The active CdtB subunit is functionally and structurally homologous to mammalian DNase I [1,2] and has to be translocated into the nucleus to be efficient [3]. It is important to note that the cytolethal distending toxin CDT should not be confused with the structurally and functionally different binary CDT toxin secreted by *C. difficile* [4].

**Figure 1:**
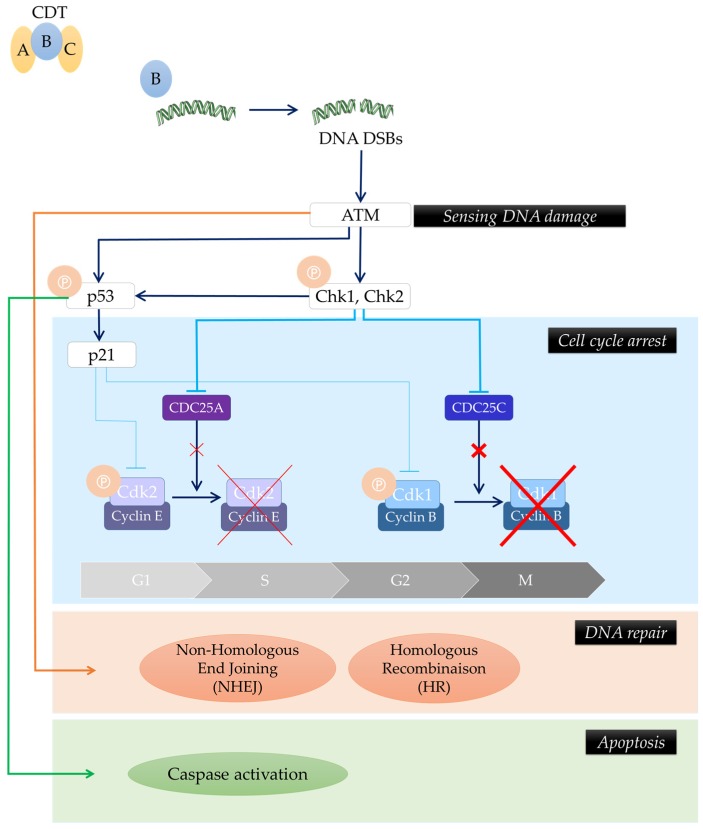
Cytolethal distending toxin (CDT)-producing bacteria induce the DNA Damage Response (DDR). CdtB induces DNA double-strand breaks (DSBs). As a result of DSBs, DNA damage response (DDR) is activated. This response is mediated by ATM (Ataxia telangiectasia mutated), leading to cell cycle arrest and initiation of DNA repair via homologous recombination (HR) and non-homologous end-joining (NHEJ) mechanisms. In some cases, the DDR system fails to properly repair DNA damage, leading to cell death by apoptosis or to long-term cell cycle arrest (known as senescence). A, B and C represent the CDT subunits (Adapted from [31]).

**Figure 2:**
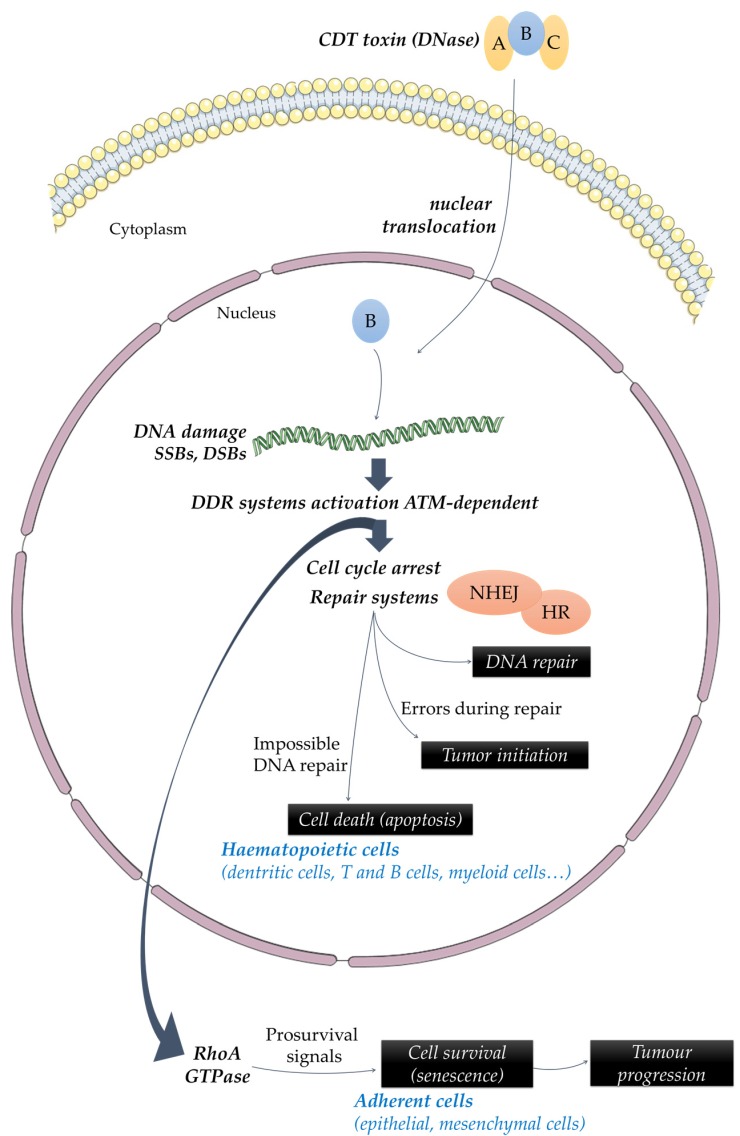
Impact of CDT-producing bacteria on cellular physiology. As a result of DSBs caused by CDT-intoxication, DNA repair mechanisms are activated, among them, DNA damage response (DDR) including homologous recombination (HR) and non-homologous end-joining (NHEJ) mechanisms. In some cases, the DDR system fails to properly repair DNA damage, leading to cell death by apoptosis in hematopoietic cells. In adherent cells, the presence of pro-survival signals (RhoA GTPase and p38) leads to cell cycle arrest and senescence. Errors made during DNA repair could favor tumor initiation whereas a senescent state could play a role in tumor progression. A, B and C represent the CDT subunits.

Owing to its DNase activity, CDT induces DNA damage. Low doses of toxin (50 pg/mL) are sufficient to induce single-strand breaks (SSBs) 3 h to 6 h post-intoxication, which are then converted into double-strand breaks (DSBs) during the S-phase of the cell cycle [5]. As a result of SSBs and DSBs, the DNA damage response (DDR) is activated [6,7]. The DDR activated as a result of CDT intoxication is the same that is activated following ionizing-radiation DSB [8] leading to an ATM (Ataxia telangiectasia mutated)-dependent cell cycle arrest at G2/M and/or G1/S transition, and initiation of DNA repair (Figure 1) [9,10,11,12], making CDT an inhibitory cyclomodulin [13,14]. This cell cycle arrest often renders different cell types involved in wound healing such as fibroblasts, keratinocytes, endothelial and epithelial cells unable to proliferate and could be responsible for a lack of tissue repair [6,15,16]. Multiple repair systems are then simultaneously activated in response to CDT-induced DNA damage: homologous recombination (HR) and non-homologous end-joining (NHEJ) mechanisms [8]. In some cases, the DDR system fails to properly repair DNA damage, leading to cell death by apoptosis or to a long-term cell cycle arrest (senescence) [12,17]. Cell fate following CDT infection seems to be cell-type dependent [6]. Indeed, epithelial and mesenchymal lineages mainly undergo cell cycle arrest [6,9,14,18,19,20], whereas hematopoietic lineages would mainly rapidly move towards apoptosis after a brief cell cycle arrest (Figure 2) [6,21,22]. The fate of cells could be explained by the activation of survival signaling pathways in adherent cells (such as epithelial and mesenchymal cells) by RhoA GTPase and p38 [23,24]. Another explanation of these different outcomes could be a greater susceptibility of hematopoietic lineages to CDT intoxication. Indeed, intoxication of human T lymphocytes with purified CDT revealed that those cells where more sensitive to the action of the toxin than HeLa cells [25]. Furthermore human T lymphocytes, contrary to epithelial lineages cells, did not present morphological alterations such as cytoplasmic elongation and distension [25]. Some authors have also hypothesized that hematopoietic lineage cells would present a sensitivity to another enzymatic activity carried out by the toxin [26,27]. One group has reported that CDT might be able to harbor a phosphatase activity, demonstrated in vitro [26]. However, if CDT exerts a genotoxic effect in a broad range of cell types [12], its phosphatase activity would play a role only in certain conditions, such as in the presence of high intracellular levels of phosphatidylinositol (3,4,5)-trisphosphate (PIP_3_) [26,27]. Indeed, antigenic and mitogenic activation, which leads to clonal expansion of lymphocytes, is dependent upon increases in PIP_3_. CDT would lead to a depletion of PIP_3_ and a concomitant inactivation of the Akt pathway, which would result in cell cycle arrest and activation of the apoptotic cascade [26]. According to their particular susceptibility to CDT intoxication, lymphocytes could be the first target of CDT, and the ensuing immunomodulation could result in persistent bacterial colonization [26,28,29]. It has been shown that the DNase activity of CDT is sufficient to induce apoptosis in non-proliferating monocytes [30]. It has been considered, therefore, that nuclease activity and DSB formation are the main mechanisms involved in CDT toxicity.

## 2. Role of CDT in Inflammation, Modulation of Immune Response and Tissue Damage

CDT is capable of inducing the release of pro-inflammatory compounds in cultured cells and in vivo. Cultured cells infected with CDT-producing bacteria present an altered cytokine expression pattern characterized by high levels of pro-inflammatory mediators (such as IL-1β, IL-6, IL-8) [17,32,33]. This altered pattern of cytokine secretion is also observed in wild-type mice, with the production of high levels of IFNγ and in contrast a decrease in anti-inflammatory cytokines such as IL-10 [28]. The antibody production pattern of CDT-infected mice is also modulated since they produce more IgG2a, IgG2c (T_h_1 associated) and IgG1 (T_h_2 associated) [28,29,34]. It has also been demonstrated that chancroid, periodontitis and campylobacteriosis patients exhibit neutralizing antibodies against CDT [35,36,37,38]. However, the correlation between the presence of CDT antibodies with severity of the disease and the role of this immune response has not yet been established.

Ando-Suguimoto et al. demonstrated that CDT inhibits macrophage phagocytosis allowing CDT-producing bacteria to proliferate [39]. CDT can also induce apoptosis in several cell lineages such as hematopoietic cells. In particular, it has been shown that T and B cells are highly susceptible to undergo apoptosis following CDT intoxication [6,22]. Dendritic cells are also subject to apoptosis following intoxication with CDT. Interestingly, it seems that the differentiation status of the cells can also influence the effects of CDT. A study on dendritic cells showed that only immature cells undergo apoptosis, illustrating a kind of immune-evasion strategy by CDT-producing pathogens [7].

Even if the sensitivity of immune cells to the action of CDT has been shown only in experimental models, it is tempting to speculate that the interference of the toxin with host defenses may impact human disease. Indeed, the resulting immunosuppression could favor bacterial growth and, as a consequence, the persistence or aggravation of chronic lesions such as chancroid lesions of *H. ducreyi* or diarrhea caused by *C. jejuni* (see below) [40].

**Figure 3:**
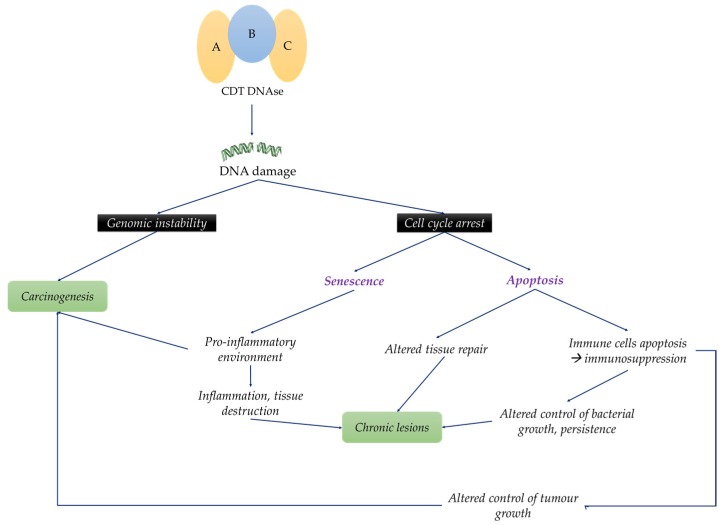
Impact of CDT-producing bacteria on infected host CDT by inducing DNA damage modifies host-physiology leading for example to a relative immunosuppressive environment, a pro-inflammatory environment or an arrest of the cell cycle. The combination of these alterations explains clinical manifestations associated to CDT-infection, mainly chronic lesions and carcinogenesis. A, B and C represent the CDT subunits.

Thus, CDT is able to modify host-cell physiology. CDT-induced immunomodulation, apoptosis or inhibition of cellular proliferation, lead to a pro-inflammatory environment, an absence of control of bacterial proliferation and a lack of tissue regeneration. This could explain several effects of the CDT-intoxication such as tissue lesions, slow healing, chronic wounds and carcinogenesis (Figure 3 and Table 1).

## 3. CDT-Producing Bacteria in Diseases

### 3.1. Camplylobacter jejuni Producing CDT and Diarrhea 

*C. jejuni* is a bacterium responsible for gastrointestinal infections, from a mild diarrhea to an acute enteric illness (pus, mucus and blood in stools) caused by inflammation of the intestinal mucosa [42]. Genes encoding CDT are found in roughly 90% of *C. jejuni* strains [43,44,45]. Several works have studied the potential role played by CDT in *C. jejuni*-related diseases. Fox et al. showed that in wild-type mice, CDT is responsible for long-term gut colonization [46]. However, they did not observe any difference in colonization of NF-κB-deficient mice by CDT-producing *C. jejuni* and an isogenic mutant in which *cdtB* has been inactivated by insertional mutagenesis. This suggests that CDT could play a role in the ability of *C. jejuni* strains to escape host immune surveillance in an NF-κB-dependent manner, leading to a long-term colonization of the host [46]. Other authors have demonstrated that CDT plays a role in the cytotoxicity and invasiveness of *C. jejuni* strains in the intestinal epithelium of immunodeficient (SCID) mice [47]. Finally, CDT promotes inflammation by inducing the production of inflammatory chemokines/cytokines such as IL-8 by intestinal epithelial cells in vitro [32]. Since IL-8 is responsible for the recruitment of polymorphonuclear neutrophils in the intestinal mucosa, leading to an increase of intestinal permeability [48], we can speculate that CDT-induced IL-8 might impair intestinal barrier integrity. Furthermore, *C. jejuni* CDT toxin is able to induce cell cycle arrest in epithelial cells [14]. We can thus hypothesize that such a cell cycle arrest in intestinal epithelial cells could impair the intestinal epithelium renewal and might contribute to decrease epithelial barrier function, and nutrient absorption. However, more data, especially in animal models are needed to fully demonstrate the implication of CDT in *C. jejuni*-induced intestinal diseases.

### 3.2. Haemophilus ducreyi Producing CDT and Chancroid Lesions 

*H. ducreyi* is a pathogen responsible for the formation of chancroid lesions, a genital ulcer disease. *H. ducreyi* has also been described as responsible for non-genital chronic skin ulcerations [49]. An epidemiological study has revealed that over 80% of clinical strains of *H. ducreyi* produce CDT [50]. In vivo studies consisting of intradermal inoculations of *H. ducreyi* strains in rabbits, showed that CDT does not seem to be required for early stage of dermal lesions formation [51,52], but contributes to the development of ulcers [53].

CDT induces cell cycle arrest in G2/M of several cell types including epithelial cells, keratinocytes and fibroblasts [6,9]. An in vitro model showed that this blockage of cell cycle affects the proliferation and the survival of cells involved in wound healing, preventing tissue regeneration and repair [15]. Furthermore, the toxin inhibits the proliferation of human T and B cells in vitro, and consequently immunoglobulin production [40]. We can then hypothesize that this immunosuppressive effect of CDT could facilitate bacterial growth and consequently the persistence of chancroid lesions and tissue damage.

### 3.3. Aggregatibacter (Actinobacillus) actinomycetemcomitans Producing CDT and Periodontitis

*A. actinomycetemcomitans* is a bacteria frequently associated with periodontitis [54], and numerous studies have found that the majority of these strains harbor CDT (from 66% to 86% depending on the study) [50,54,55,56]. Periodontitis is an inflammatory state of the tooth-supporting structures (gingiva, alveolar bone, periodontal ligament and cementum). If left untreated, the disease progresses to bone destruction and subsequent tooth loss. Some authors identified the presence of CDT as a factor of aggressiveness of the disease [57]. Indeed, by its ability to block the cell cycle and hence to inhibit the proliferation of human cells in culture (periodontal ligament cells, gingival fibroblasts) [16], CDT could prevent tissue regeneration. But the role of CDT in this disease does not seem to be limited to a lack of tissue repair. Under the action of CDT, human T and B cells undergo in vitro cell cycle arrest, leading to an alteration of immunoglobulin production [25,58]. This illustrates how CDT could prevent the immune system from protecting tissue against bacterial attack, which results in a failure of bacterial clearance. 

Works performed using human peripheral blood mononuclear cells (PBMCs) have shown that CDT is also responsible for induction of pro-inflammatory cytokine production by these cells [59]. This CDT-induced modulation of host immunity and inflammatory responses could explain at least in part in the bone destruction observed in periodontitis. Indeed, pro-inflammatory mediators activate the receptor activator of nuclear factor kappa-B (RANK) ligand (RANKL) pathways and so stimulate bone resorption [60]. RANK is expressed on osteoclast progenitor cells while RANK ligand (RANKL) is present at the surface of osteoblasts, hematopoietic bone marrow stromal cells as well as T cells [61,62]. When RANKL binds to RANK, it leads to osteoclast maturation. It has been shown that CDT upregulates RANKL in Jurkat T-cells [63]. This results in an increased amount of RANKL binding to RANK, which may favor the differentiation of cells into osteoclasts. In doing so, CDT could promote the destruction of tissue instead of its synthesis [63,64]. More recently, Damek-Propawa et al. also demonstrated that CDT is involved in the remodeling of adherens junctions in human gingival explants and human gingival epithelial cells [65]. These junctions are essential for maintaining the integrity and barrier function of gingival epithelium on the comforting structures of the teeth. Finally, a study using rats showed that inoculation of CDT in gingival tissue triggered cell cycle arrest of cells, resulting in tissue abrasion, supporting the hypothesis of an implication of CDT in periodontal disease [66].

## 4. Potential Involvement of CDT in Cancers

As discussed above, CDT toxin is probably involved in the severity of several human diseases caused by bacteria. However, the role played by CDT is probably larger and many properties of this toxin support its involvement in human cancers.

As previously mentioned, CDT toxin presents a DNase activity, leading to DNA damage. DNA damage are lesions associated to cancer and it is tempting to speculate that CDT-induced DNA damage might be involved in cancer promotion/progression. Furthermore, it has been shown in vitro, that chronic intoxication with sublethal doses of CDT does not cause cell death or cell cycle arrest. Instead, it induces limited DNA damage, increases the frequency rate of mutations and consequently, results in chromosomal instability [67]. This genomic instability leads to activation of DDR systems and pro-survival signals enhancing anchorage-independent growth [67], which are traits of malignant transformation. These observations support the notion that CDT might promote tumor initiation and progression.

Another property of CDT toxin supporting its role in cancer is the pro-inflammatory action of this toxin demonstrated in vitro as well as in vivo. Indeed, it is well known that a pro-inflammatory environment sustains tumor survival, proliferation and progression (via promoting angiogenesis and metastasis) [68]. Furthermore, it should be noted that some cell types undergo cellular senescence following CDT-intoxication, and consequently harbor a senescence-associated secretory phenotype (SASP) [17]. This SASP is characterized by secretion of a large panel of growth factors and pro-inflammatory cytokines. Interestingly, it has been shown that senescent cells may promote, *via* the SASP, the survival and the proliferation of transformed cells [69]. Therefore, we can imagine that CDT could also favor tumor development by triggering a senescent-associated secretome in infected cells. Thus, the capacity of CDT to create a pro-inflammatory and or a growth factor enriched micro-environment could then be another way to promote carcinogenesis.

Finally, CDT could be also involved in tumorigenesis by modulating immune response. We previously discussed how immune cells are particularly sensitive to CDT-infection, making them a prime target of CDT. However, these immune cells, especially T and B cells, are major actors of anti-tumor immune response by targeting and lysing tumor cells [70]. So, it is tempting to speculate that the immunosuppression induced by CDT will lead to a lack of control of tumor growth, favoring cancer development.

Altogether, DNA damage, pro-inflammatory environment or immunosuppression induced by CDT support the notion that CDT might promote tumor initiation and/or progression. Interestingly, some in vivo experimental data as well as epidemiological studies support this hypothesis. *H. hepaticus* is a pathogen of mice, known to cause chronic active hepatitis and hepatocarcinoma [71]. In an IL10^−/−^ mouse model, CDT-producing *H. hepaticus* induces an IgG1 and IgG2c immune response, whereas *H. hepaticus* defective for CDT production fails to induce a strong immune response, demonstrating the immunomodulatory role of CDT [29]. CDT has also been shown to be responsible for the genotoxicity induced by *H. hepaticus* on cultured cells [72]. More importantly, experiments performed using inbred A/JCr mice have demonstrated the implication of CDT in the development of hepatic dysplasia [73]. Indeed, 80% of mice infected with CDT-producing *H. hepaticus* developed early dysplastic lesions whereas none of the CDT-mutant infected mice developed hepatocellular dysplasia [73]. However, it should be noted that CDT did not influence the severity of *H. hepaticus*-induced hepatitis, although CDT-producing strains were associated with an upregulation of pro-inflammatory mediators, an activation of the NF-κB pathway and an increase of hepatocyte proliferation [73]. Taken together, these data suggest the immunomodulatory role of CDT and its implication in the development of dysplastic lesions.

Interestingly, a recent study demonstrated that CDT-producing *E. coli* are frequently found to be associated with human colorectal cancer biopsies (15.8% in tumor biopsies *vs* 0% in control tissue; *p* < 0.02) [74]. Considering the previous findings about CDT-producing *H. hepaticus* and hepatic dysplasia, it is tempting to imagine a possible pro-carcinogenic effect of the colorectal cancer-associated CDT-producing *E. coli.* Recently a study performed in vitro using human colon epithelial cells suggested that CDT derived from *E. coli* might play a potential role in colorectal cancer promotion but not initiation. Data reported in this study show that upon CDT exposure, only cells with a silenced p53 are able to proliferate without attachment to the extracellular matrix [75]. However, further studies are needed to confirm if CDT is effectively involved in colorectal carcinogenesis.

## 5. Conclusions

CDT is a toxin widely present in pathogenic bacteria. In vitro studies have demonstrated that it can profoundly alter eukaryotic cellular physiology. Interestingly, several in vivo studies tend to demonstrate how some diseases induced by CDT-producing bacteria could be linked, at least in part, to the deleterious effect of the toxin. Modulation of the host immune response leading to inflammation, persistence of the pathogens and chronic wounds, is likely an important factor in the resulting disease. The effect of CDT on human physiology is probably greater since some studies have also suggested that CDT-producing bacteria could be involved in cancer.

## Figures and Tables

**Table 1:** Possible contribution of select CDT-producing species in pathology (adapted from [12,41]).

Select CDT-Producing Species	Pathology	Possible Contribution of CDT
*Aggregatibacter actinomycetemcomitans*	Aggressive periodontal disease	Aggressiveness of disease
*Campylobacter jejuni*	Inflammatory diarrhea	Prolongation of symptoms, Persistence of infection
Colorectal cancer-associated *Escherichia coli*	Colorectal cancer	Potential promotion of cancer initiation or progression, Potential promotion of carcinogenesis
*Haemophilus ducreyi*	Chancroid lesions	Development of ulcers, Persistence of lesions
*Helicobacter hepaticus*	Hepatitis, hepatocarcinoma (in mice)	Contribution to carcinogenesis (inflammation-induced carcinogenesis)
*Salmonella enterica* serovar Typhi	Diarrhea	Prolongation of symptoms, Persistence of infection
*Shigella dysenteriae/boydii*	Diarrhea	Role to be defined
